# Increased mtPDH Activity Through Antisense Inhibition of Mitochondrial Pyruvate Dehydrogenase Kinase Enhances Inflorescence Initiation, and Inflorescence Growth and Harvest Index at Elevated CO_2_ in *Arabidopsis thaliana*

**DOI:** 10.3389/fpls.2016.00095

**Published:** 2016-02-12

**Authors:** Sarathi M. Weraduwage, Malgre C. Micallef, Elizabeth-France Marillia, David C. Taylor, Bernard Grodzinski, Barry J. Micallef

**Affiliations:** ^1^Department of Plant Agriculture, University of GuelphGuelph, ON, Canada; ^2^National Research Council of CanadaSaskatoon, SK, Canada

**Keywords:** *Arabidopsis*, elevated carbon dioxide, mitochondrial pyruvate dehydrogenase kinase, inflorescence growth, silique and seed production, harvest index, source-sink relationships

## Abstract

Mitochondrial pyruvate dehydrogenase (mtPDH) is a key respiratory enzyme that links glycolysis and the tricarboxylic acid cycle, and it is negatively regulated by mtPDH kinase (mtPDHK). *Arabidopsis* lines carrying either a constitutive or seed-specific antisense construct for mtPDHK were used to test the hypothesis that alteration of mtPDH activity in a tissue- and dosage-dependent manner will enhance reproductive growth particularly at elevated CO_2_ (EC) through a combined enhancement of source and sink activities. Constitutive transgenic lines showed increased mtPDH activity in rosette leaves at ambient CO_2_ (AC) and EC, and in immature seeds at EC. Seed-specific transgenic lines showed enhanced mtPDH activity in immature seeds. A strong relationship existed between seed mtPDH activity and inflorescence initiation at AC, and at EC inflorescence stem growth, silique number and seed harvest index were strongly related to seed mtPDH activity. Leaf photosynthetic rates showed an increase in rosette leaves of transgenic lines at AC and EC that correlated with enhanced inflorescence initiation. Collectively, the data show that mtPDHK plays a key role in regulating sink and source activities in *Arabidopsis* particularly during the reproductive phase.

## Introduction

Less than predicted enhancements in photosynthetic capacity and yield in plants under elevated CO_2_ (EC) have been attributed to insufficient sink capacity and end-product inhibition of photosynthesis (Clough et al., 1981; Arp, 1991; Stitt, 1991; Ainsworth et al., 2004; Long et al., 2004, 2005; Rogers et al., 2004; Sharkey et al., 2004; Ainsworth and Rogers, 2007; Leakey et al., 2009). Therefore, investigation of potential physiological and biochemical processes through which sink strength can be enhanced to improve growth and productivity in plants under EC remains essential. Rarely has dark respiration *per se* been considered a promising target to increase sink activity and productivity, owing to its role in catabolic processes (Drake et al., 1999; Sharma-Natu and Ghildiyal, 2005; Long et al., 2006). Indeed, studies carried out at ambient CO_2_ (AC) in starch-storing plants show that decreased respiratory CO_2_ release correlates with enhanced productivity (Wilson and Jones, 1982; Winzeler et al., 1989).

With the advent of biotechnological tools, most attempts to modify respiratory genes throughout the past decade and a half have focused on suppression of glycolytic and tricarboxylic acid (TCA) cycle gene expression (Knowles et al., 1998; Yui et al., 2003; León et al., 2007; Nunes-Nesi et al., 2007; Sienkiewicz-Porzuceka et al., 2010). Interestingly, when citrate synthase and the mitochondrial pyruvate dehydrogenase (mtPDH) enzyme complex activities were enhanced, significant positive results on growth were observed at AC, although effects on seed production and harvest index were not reported (Zou et al., 1999; Koyama et al., 2000; Marillia et al., 2003). These data provide evidence that dark respiration plays an important role in anabolic processes in plants and in maintaining sink activity and source-sink balance.

The mtPDH complex is a key enzyme in dark respiration that catalyzes the oxidative decarboxylation of pyruvate to acetyl-CoA and NADH with the evolution of CO_2_ (Reid et al., 1977; Budde and Randall, 1990; Gemel and Randall, 1992; Buchanan et al., 2000; Mooney et al., 2002). This enzyme catalyzes an essentially irreversible reaction that is regulated post-translationally through the negative regulator mitochondrial pyruvate dehydrogenase kinase (mtPDHK; Reid et al., 1977; Budde and Randall, 1990; Gemel and Randall, 1992; Buchanan et al., 2000; Mooney et al., 2002). The mtPDH links glycolysis and the TCA cycle, and it provides acetyl-CoA for both oxidative processes and for many anabolic processes such as fatty acid and terpenoid synthesis, thus rendering the mtPDH complex an important site for metabolic regulation (Gemel and Randall, 1992; Buchanan et al., 2000).

Transgenic *Arabidopsis* lines having constitutive antisense expression of mtPDHK showed elevated mtPDH complex activity in leaves, greater activity of leaf citrate synthase, enhanced respiratory CO_2_ release at 25°C, early transition from a vegetative to a reproductive stage, greater rates of incorporation of ^14^C-pyruvate into seed fatty acids, and increased 100-seed weight and oil weight at AC (Zou et al., 1999; Marillia et al., 2003), suggesting that these transgenic lines have greater sink strength. Dahal et al. (2014) provided a preliminary report indicating that these constitutive lines show enhanced seed and oil yield particularly at EC, although the developmental basis for this yield effect was not reported. A recent whole-plant gas exchange study by Leonardos et al. (2014) that examined two of the constitutive antisense mtPDHK lines provided evidence for enhanced sink activity at EC at the inflorescence stage relative to control lines. These constitutive lines when grown at AC can show a reduction in vegetative growth (e.g., smaller rosette leaves; Zou et al., 1999; Marillia et al., 2003; Leonardos et al., 2014), suggesting that an increase in mtPDH activity through constitutive suppression of mtPDHK can have negative effects on plant growth at AC. Transgenic lines showing seed-specific expression of antisense mtPDHK showed the greatest enhancement in seed oil content (Marillia et al., 2003). Potential tissue- and dosage-effects of mtPDH activity on vegetative and reproductive growth at AC and EC in these constitutive and seed-specific transgenic lines have not been examined. In addition, while there is evidence that mtPDHK transcript levels are reduced at EC in *Arabidopsis* (Leonardos et al., 2014), the effect of EC on mtPDH activity has not been examined to date in any plant.

Given the central role of mtPDH in supplying C intermediates to anabolic processes and given the evidence that antisense suppression of mtPDHK enhances sink strength in *Arabidopsis* under AC, the *Arabidopsis* model system examined by Zou et al. (1999), Marillia et al. (2003), Dahal et al. (2014) and Leonardos et al. (2014) was used in the present study to examine both the effect of EC on mtPDH activity in leaves and immature seeds, and to determine if *Arabidopsis* with alteration of mtPDH activity show enhanced reproductive growth and plant productivity at EC owing to a combined enhancement of both source and sink activities. It was hypothesized that *Arabidopsis* lines having suppressed mtPDHK expression will show an enhancement of mtPDH activity in leaves and seeds, that mtPDH activity will be enhanced at EC, and that the greatest enhancement of reproductive growth will occur particularly at EC for lines showing seed-specific suppression of mtPDHK. To test the hypothesis above we examined vegetative and reproductive growth and seed production in *Arabidopsis* lines with altered mtPDHK expression under EC; mtPDH activity was also measured. Collectively, the data show that leaf mtPDH activity is enhanced at EC, and that mtPDHK plays a key role in regulating reproductive growth in *Arabidopsis* particularly at EC.

## Materials and methods

### Plant material

*Arabidopsis thaliana* L. ecotype Columbia untransformed wild-type plants (WT), constitutive (C1, C2, C3, C4) and seed-specific (S1, S2, S3, S4) transgenic lines containing an antisense construct for mtPDHK and their respective control lines carrying the empty vector (pBI121 and pRD400) were utilized. Lines C1, C2, C3, C4, S1, S2, S3, and S4 in the present study correspond to lines 10^4^, 3^1^, 9^5^, 5^2^, 1^1^B, 5^6^E, 5^6^J, and 10^2^A, respectively, mentioned in Zou et al. (1999), Marillia et al. (2003), Dahal et al. (2014) and Leonardos et al. (2014). Transgenic lines were previously shown to be homozygous and to contain a single copy of the antisense construct (Zou et al., 1999; Marillia et al., 2003; Leonardos et al., 2014). All transgenic lines showed suppression of mtPDHK (Zou et al., 1999; Marillia et al., 2003; Leonardos et al., 2014). The T_4_ generation was used for this study. T_4_ seeds were obtained by growing the T_3_ generation for the constitutive and seed-specific lines in the same growth chamber under control conditions at AC, and the T_4_ seeds from 3 plants were pooled to establish the seed stock for each line.

### Experimental design and growth conditions

To assess the influence of atmospheric CO_2_ levels on vegetative and reproductive growth parameters throughout the entire development period for the 11 *Arabidopsis* lines, two separate growth chamber experiments were performed over time (i.e., blocks) to account for potential chamber effects; these are identified as growth experiment 1 and growth experiment 2. Thus, the experimental design for these determinations was a split plot design using two blocks over time (*n* = 2, experimental unit = growth chamber) for each of the AC and EC treatments. The two replicates for each of the two CO_2_ treatments were performed in different growth chambers. To assess differences between lines within the split plot design for a given CO_2_ treatment, all 11 *Arabidopsis* lines were arranged within each growth chamber using a completely randomized design to minimize growth chamber effects; *n* = 10 experimental units per line, where an experimental unit = a pot with one plant.

For each experimental replicate, one growth chamber was maintained at AC (380 CO_2_), and a second chamber was set at EC (700 ppm CO_2_). An irradiance of 200 μmol m^−2^ s^−1^, a 12 h photoperiod, 20°C continuous temperature and a set point of 60% relative humidity (R.H.) were used for each growth chamber. Growth chambers belonging to the GC-20, Bigfoot series (BioChambers Inc., Winnipeg, MB, Canada) situated in the Biotron facility in the Bovey Building, University of Guelph, were used. Plants were grown in Pro-Mix BX media (not containing Mycorrhizae) in 4″ pots and fertilized with ½ strength Hoagland's solution.

### Determination of mtPDH complex activity in crude leaf and seed extracts

Leaf tissue (450 mg) devoid of midrib was harvested from leaf rosettes 2–3 h into the dark period. The harvested tissue was immediately ground to a fine powder in liquid N_2_ and transferred to a sterile 2.5 mL-Eppendorf tube with the aid of a sterile spatula. Both the Eppendorf tube and spatula were chilled by dipping in liquid N_2_ prior to use. Mortars and pestles were autoclaved and chilled prior to use. Two mL of extraction buffer (40 mM Tris buffer, pH 7.5 with 5 mM DTT) was added to the tissue for grinding. The composition of the extraction buffer was a modification of the suspension buffer used by Zou et al. (1999). The resulting homogenate was then vortexed for 1 min followed by centrifugation at 13,000 rpm for 20 min. The supernatant was then transferred to a fresh microfuge tube and placed on ice, and the green pellet was discarded. The crude extract was immediately assayed for mtPDH complex activity and inactivation by ATP. The composition of the assay buffer was modified from Zou et al. (1999) and was as follows: 100 mM Tricine (pH 7.5), 0.1 mM TPP, 0.5 mM MgCl_2_, 1.5 mM NAD^+^, 0.1 mM CoASH, 3.0 mM cysteine-HCl, 1.5 mM pyruvic acid and 300 μL of enzyme extract in a total volume of 1 mL. After the addition of pyruvate to initiate the reaction, contents were mixed using a pipette and allowed to stand for 4 min prior to determination of activity by monitoring the formation of NADH at 340 nm using a spectrophotometer (Ultraspec 2100pro, GE Healthcare, UK) connected to a water circulator set at 20°C (LAUDA K-4RD, Brinkmann Instruments, Delran, NJ). Control reactions contained all components except pyruvate. Data were plotted using SWIFT Reaction Kinetics 2.0 (Biochrom Ltd, UK), and the specific enzyme activity was determined as described by Wilson and Walker (2010). Protein levels in the crude extract was determined by measuring the absorbance at 280 and 260 nm and substituting the measurements in the Warburg-Christian formula as follows: protein concentration (mg/mL) = 1.55 × (A_280_) − 0.76 × (A_260_) (Wilson and Walker, 2010).

ATP-dependent inactivation of the mtPDH complex by mtPDHK was determined by incubating leaf extracts with 5 mM ATP with or without phosphatase inhibitors (10 mM NaVO_3_ and 50 mM NaF) in assay buffer for 6 min to allow phosphorylation of mtPDH followed by reaction initiation with or without pyruvate as described above. The % retention of activity in the presence of ATP with or without phosphatase inhibitors was used to determine the % total activity that could be attributed to the mitochondrial isozyme, since the plastidial isozyme is not deactivated by phosphorylation (Rubin and Randall, 1977; Budde and Randall, 1990; Luethy et al., 1996; Tovar-Méndez et al., 2003).

Quantification of the mtPDH complex activity in crude extracts of immature seeds and ATP-inactivation assays were carried out in a similar manner using 0.25 g of immature seeds in the mid to late maturation phase of development (Baud et al., 2008) owing to the amount of tissue required, and 1.5 mL of extraction buffer. After vortexing and the initial 20 min centrifugation step, the resulting supernatant was brownish and turbid, unlike the homogenates from leaves. This was likely a result of higher protein and starch content in seeds. In addition, there was a white layer of oil-mixed debris on the surface of the supernatant. Therefore, the supernatant was carefully removed to avoid contamination from the upper oil layer and the pellet of cell debris in the bottom. The supernatant was then re-centrifuged at 13,000 rpm for another 2 min, which yielded a small pellet and a clear, faintly yellow colored supernatant that was transferred to a fresh Eppendorf tube and immediately placed on ice. The crude extract was immediately assayed for mtPDH complex activity as described for the leaf assay, except that 50 μL of enzyme extract and 3 mM pyruvate were used. Also, 15 mM ATP was required for ATP-inactivation assays.

### Extraction and quantification of seed oil

Analyses of seed oil content were performed at The National Research Council, Saskatoon, SK, via gas chromatography. Since differences in plant productivity between WT and transgenic lines was greatest in growth experiment 1, oil and fatty acid content was analyzed from seeds collected from growth experiment 1. The content of specific fatty acids and total oil content as a percentage of seed weight was then calculated as described previously (Katavic et al., 1995; Zou et al., 1997).

### Determination of growth and developmental parameters

A comprehensive growth analysis was carried out to measure the maximum rosette radius (Boyes et al., 2001), total number of rosette leaves, number of days required for visual appearance of the inflorescence (inflorescence initiation), the total length of primary (1°), secondary (2°), tertiary (3°) inflorescence axes, and inflorescence axes arising from the rosette leaf axils, total inflorescence span, number of siliques borne in the different inflorescence axes and the total number of siliques produced by the entire plant (Supplementary Figure 1). Measurements were taken at 4 different time points of the life cycle. Mature seeds were harvested and allowed to dry to determine the total seed weight and the average weight of 100 seeds and the number of seeds was calculated as follows: total number of seeds per plant = [total seed weight/100-seed weight] × 100. Using the data on silique and seed production, the average number of seeds per silique was determined. Harvest index (HI) in terms of seed and oil production was calculated as follows: HI (seed) = [total seed weight (g/plant)/total above ground dry weight (g/plant)] × 100; HI (oil) = [total oil weight (g/plant)/total above ground dry weight (g/plant)] × 100.

### Measurements of net CO_2_ exchange rate

Net CO_2_ exchange rate in the light and dark for individual rosettes leaves at 49–53 days after seed sowing (DAS) was measured at AC and EC using a LI-6400 portable gas exchange system (LI-COR Biosciences, Inc., Lincoln, NB) with an attached *Arabidopsis* chamber (6400-15). The light and dark net C assimilation rates were determined during the corresponding 12 h light and 12 h dark periods, respectively. Set up, calibration and measurements were carried out according to the LI-6400 user manual. The conditions in the *Arabidopsis* chamber included a leaf temperature of 20°C, leaf fan speed set to slow, [CO_2_]of either 380 (AC) or 700 ppm (EC), 60% R.H., and the vapor pressure deficit between chamber and leaf air spaces was maintained between 0.5 and 1 kPa. The flow rate was set at 300 μmol s^−1^, and the leaf area set to 0.785 cm^2^. Illumination was provided by growth chamber lights set at 200 μmol m^−2^ s^−1^.

### Statistical analyses

Statistical analyses were carried out using SPSS 18 (IBM Corporation, NY, NY). Data from the two growth experiments for each parameter were combined and treatment and variety effects were compared using a general linear model and data were subjected to two-way multivariate analysis of variance (MANOVA). Based on the significance of the multivariate test, the univariate main effects were investigated followed by a least significant difference (LSD) test at α = 0.05.

In order to (1) to establish the relationship between various growth and productivity parameters at a given CO_2_ treatment, (2) to establish the relationship between mtPDHK expression or mtPDH activity and specific growth and productivity parameters at a given CO_2_ treatment, and (3) to allow a comparison between responses at ambient vs. elevated CO_2_, multivariate principal component analyses were performed separately on data collected from AC and EC grown plants. Linear correlation between certain parameters was tested using the Pearson correlation coefficient (r) followed by a one-tailed *t*-test for significance.

## Results

### Activity of mtPDH in rosette leaves and immature seeds

**Figure 1:**
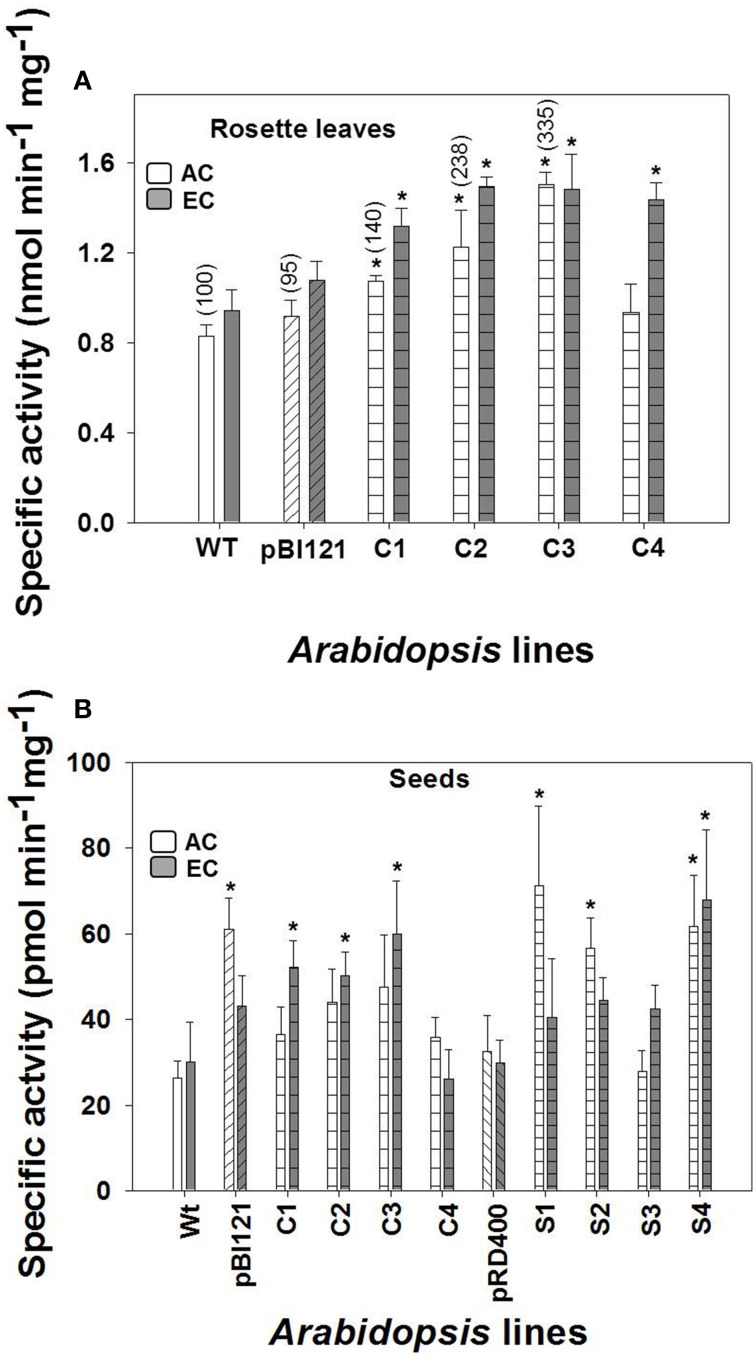
**Maximal leaf and seed mtPDH complex activity in ***Arabidopsis*** lines grown at either AC or EC**. Specific enzyme activity of mtPDH in the rosette leaf of constitutive lines **(A)**, and specific enzyme activity of mtPDH in seeds at the mid to late maturation phase of development for all lines at AC and EC **(B)** is presented. Bracketed values in **(A)** represent relative leaf mtPDH activities given in Zou et al. (1999) determined using isolated mitochondrial preparations for lines grown under AC at 23°C and assayed at 28°C. CO_2_ treatments: 380 ppm (AC, white bars), 700 ppm (EC, gray bars). Asterisks (^*^) indicate results significantly different from wild-type (WT) at α = 0.05 when compared within each CO_2_ treatment. Values represent the mean±SE; *n* = 3−7 plants per line in **(A)**, and *n* = 5−8 plants in **(B)**. To convert the activities in nmol (or pmol) min^−1^ mg^−1^ protein to nmol min^−1^ g^−1^ fresh weight to allow a direct comparison of activities, the following conversion factors are used: (1) rosette leaf, 32 × nmol min^−1^ mg^−1^ protein = nmol min^−1^ g^−1^ fresh weight; and (2) seed, 0.3 × pmol min^−1^ mg^−1^ protein = nmol min^−1^ g^−1^ fresh weight.

Compared to WT, the constitutively-suppressed lines C1, C2, and C3 showed a significantly higher specific mtPDH activity in rosette leaves at AC, and all constitutively-suppressed lines had a significantly higher mtPDH activity than controls at EC (Figure 1A). The order of rosette mtPDH complex activity at AC in constitutive lines was C3>C2>C1>C4> pBI121>WT (Figure 1A). This is the same order of rosette mtPDH activities found by Zou et al. (1999) when these lines were assayed using isolated mitochondria (Figure 1A, bracketed values). While C1 and C2 showed moderate increases in rosette mtPDH activity of 30 and 48% respectively, C3 exhibited the highest increase in activity of 81% relative to WT at AC (Figure 1A). At EC, mtPDH activity in rosettes was 44–58% higher in constitutive lines, with C2 (58%) and C3 (57%) having the highest activity relative to WT (Figure 1A). At EC, the order of rosette mtPDH complex activity was C2>C3>C4>C1>pBI121>WT (Figure 1A). Activity of mtPDH in rosette leaves was greater at EC compared to AC except for line C3 (Figure 1A).

At AC, the seed-specific lines S1, S2, and S4 exhibited seed mtPDH activities that were 169, 114, and 134% higher, respectively, than WT (Figure 1B). At EC, seed mtPDH complex activity was highest in the seed-specific line S4, where it was 126% higher than WT (Figure 1B) and constitutive lines C1, C2, and C3 showed activities that were 74, 67, and 100% higher, respectively, than WT. In contrast to rosette leaves, there was no consistent increase in seed mtPDH activity at EC compared to AC, except for the constitutively-suppressed lines C1, C2, and C3 (Figure 1).

When protein extracts were first incubated with 5 mM ATP (rosette leaf assays) or 15 mM ATP (seed assays) for 6 min, 95% of PDH activity was removed. The residual PDH activity was removed from the leaf and seed extracts by incubating with ATP and phosphatase inhibitors (10 mM NaVO_3_ and 50 mM NaF) (data not shown). ATP-inactivation analysis showed that essentially all PDH activity measured in crude extracts was mitochondrial and that no contaminating plastidial PDH (plPDH) activity was present, based on the finding that plPDH is not inactivated by phosphorylation like mtPDH (Rubin and Randall, 1977; Budde and Randall, 1990; Luethy et al., 1996; Tovar-Méndez et al., 2003). The rosette and seed mtPDH activities, when based on units of nmol min^−1^ g^−1^ fresh weight are the same order of magnitude (see Figure 1 legend).

### Vegetative and reproductive growth and plant productivity in *Arabidopsis* with enhanced mtPDH activity

A two-way multivariate ANOVA analysis showed significant multivariate main effects for CO_2_ treatment and *Arabidopsis* lines at both AC and EC, and there was also a significant interaction between CO_2_ treatment and *Arabidopsis* lines (Supplementary Table 1). These significant main effects and CO_2_-*Arabidopsis* line interactions were followed up by univariate analysis of variance and mean comparisons, correlation analysis, and principle component analysis to determine the impact of CO_2_ treatment and *Arabidopsis* lines (with altered mtPDH activity) on the many growth and productivity parameters that were measured and results are presented in the following sections. The term “transgenic” in the Results refers to the mtPDHK antisense lines, and it does not refer to the transgenic lines carrying empty vector: pBI121 (constitutive) and pRD400 (seed-specific).

#### Vegetative growth and net C exchange

**Figure 2:**
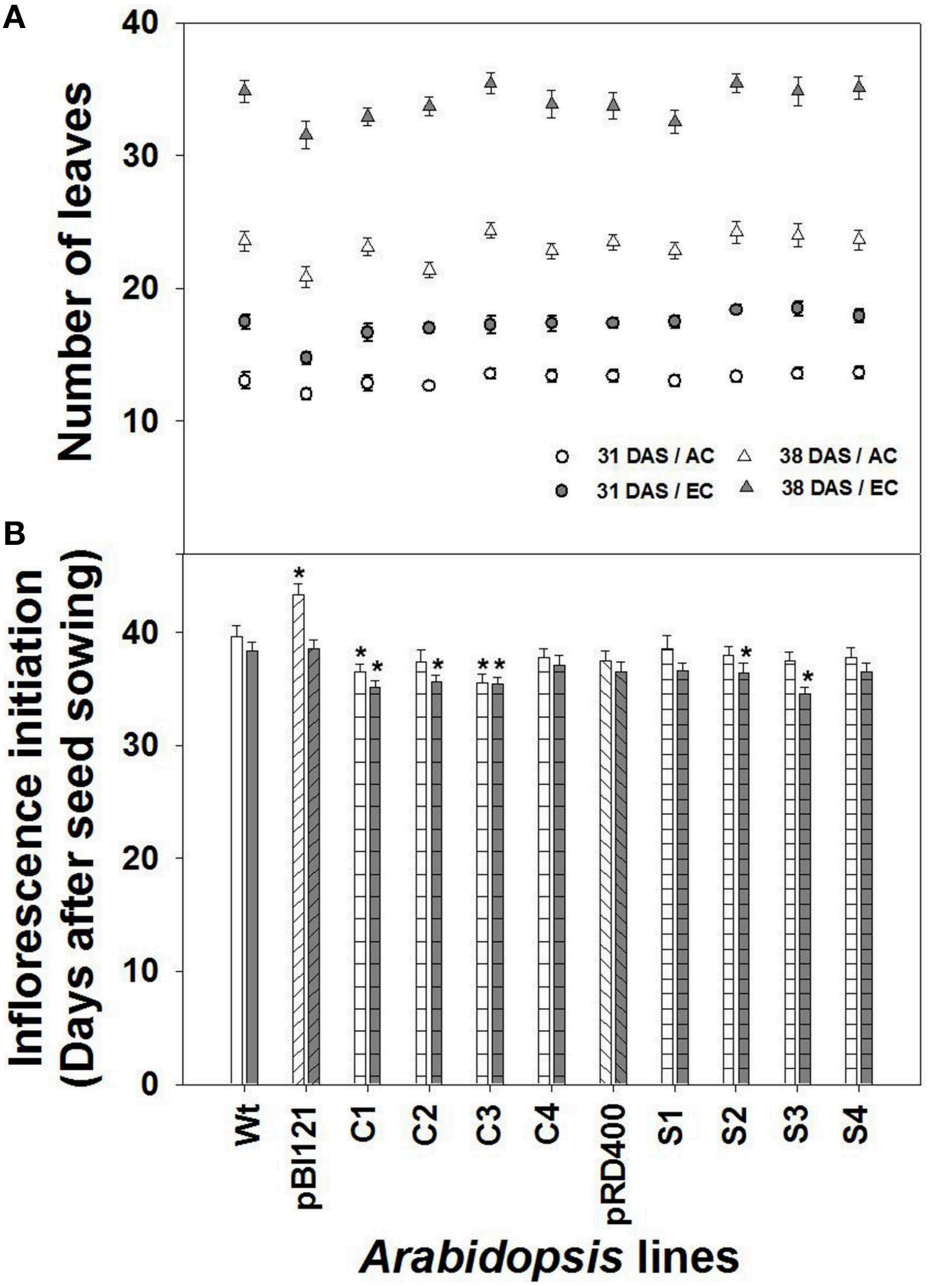
**Comparison of rosette leaf number and inflorescence initiation over time in ***Arabidopsis*** lines grown at either AC or EC**. Total rosette leaf numbers at 31 and 38 DAS **(A)** and the number of days required for visible inflorescence initiation **(B)** are presented. In **(A)**, the data at 38 DAS represents the final leaf numbers at the end of the vegetative phase. CO_2_ treatments: 380 ppm (AC, open circles or triangles), 700 ppm (EC, closed circles or triangles). Asterisks (^*^) indicate results significantly different from wild-type (WT) at α = 0.05. Values represent the mean ± SE; *n* = 10 plants per line.

The number of rosette leaves (Figure 2A), rosette size, and the rate of rosette growth (Supplementary Figures 2A–D) were greater at EC than at AC for all lines. The number of leaves produced by transgenic lines was similar to that of WT at AC and EC (Figure 2A). While no significant increase in rosette size at EC was found for seed-specific transgenic lines compared to WT, constitutive line C2 showed significantly larger rosettes during the vegetative growth phase both at AC and EC (Supplementary Figure 2).

**Figure 3:**
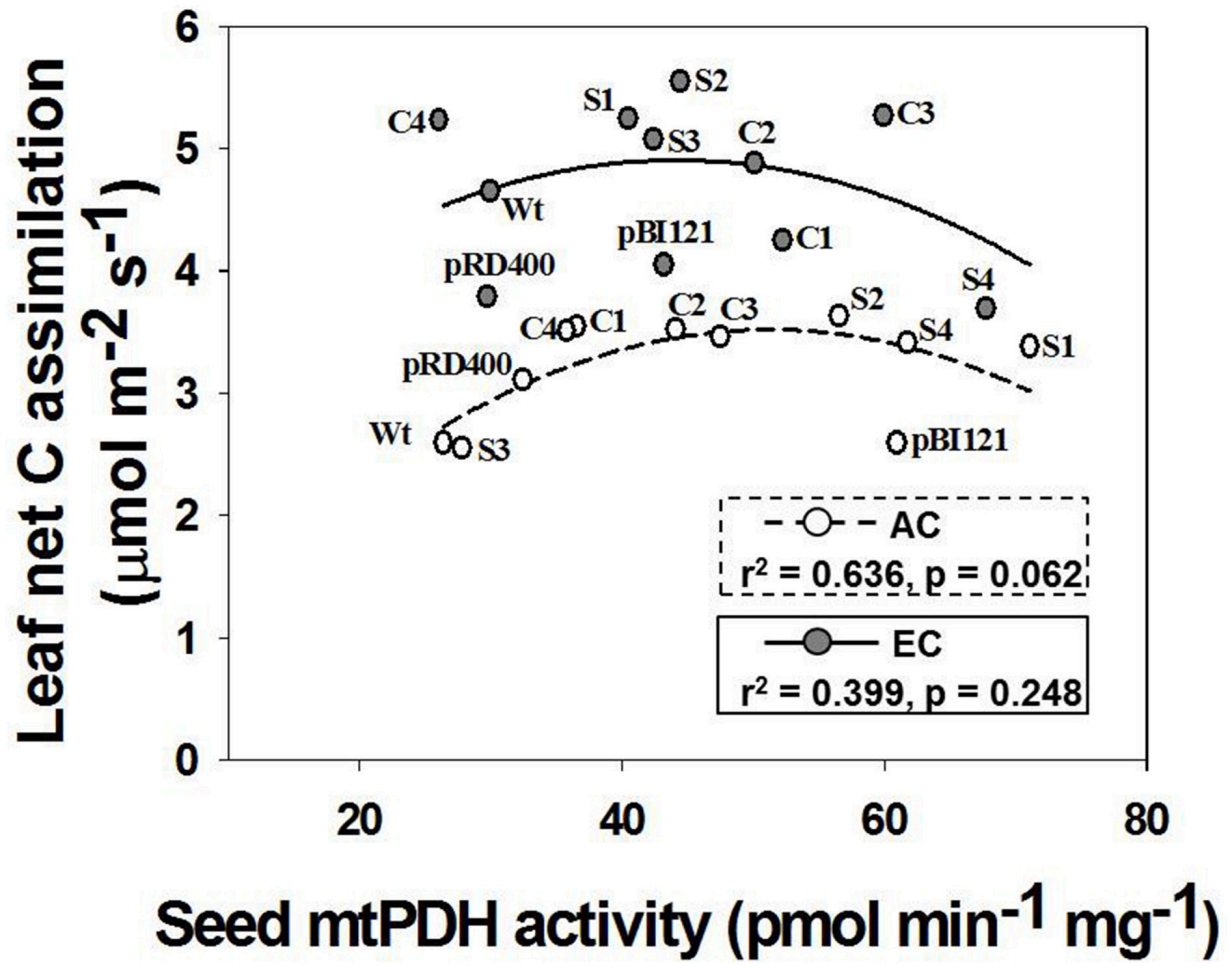
**Daytime net C assimilation in rosette leaves of ***Arabidopsis*** lines grown at either AC or EC**. Quadratic regressions between rosette net C assimilation rate and seed mtPDH activity for all *Arabidopsis* lines is presented along with the r^2^ value, significance levels, and lines of best fit. Photosynthetic rates of rosette leaves were determined at 49–53 DAS and plants were already flowering at this stage. CO_2_ treatments: 380 ppm (AC, open circles/dashed line), 700 ppm (EC, closed circles/solid line). Values for photosynthesis represent the mean; *n* = 5−7 plants per line.

Leaf C fluxes were measured for individual rosette leaves at 49–52 DAS (Figure 3). The rosette leaves were fully expanded at this developmental stage for all lines, and the 1° inflorescence was visibly initiated in all lines and actively growing. Thus, the leaf was fully functional as a source tissue at this stage, minimizing effects of leaf maturity on gas exchange, and a reproductive sink was established. Suppression of mtPDHK in transgenic lines had no negative effect on photosynthesis in rosette leaves of *Arabidopsis* lines under both CO_2_ treatments, but instead it seemed to increase area-based photosynthetic rates in transgenic lines, and particularly for constitutive and seed-specific lines at AC (Figure 3). Furthermore, the rates of photosynthesis at EC were higher than the rates at AC (Figure 3). Investigation of whether altered seed mtPDH activity in the established inflorescences/sink tissue correlated with leaf photosynthesis, revealed a strong quadratic relationship between seed mtPDH activity and photosynthetic rate at AC. The highest rates of photosynthesis in the rosette leaves were found for transgenic lines showing an intermediate seed mtPDH activity at both AC and EC. As reported by Leonardos et al. (2014) for constitutive transgenic lines C1 and C2, the night time net CO_2_ exchange rates were not significantly different between lines at either AC or EC, and the rates at EC were generally higher than at AC (data not shown).

#### Reproductive growth and plant productivity

##### Inflorescence initiation and size

Constitutive lines C1 and C3, showed early inflorescence initiation at AC (Figure 2B). At EC, inflorescence initiation in lines C1, C2, C3, S2 and S3 occurred significantly earlier than WT. Overall early inflorescence initiation was most consistent in constitutive transgenic lines (Figure 2B). Usually, leaf number and time for flower initiation is negatively correlated in *Arabidopsis* (Takahashi and Morikawa, 2014). However, comparison of leaf number between the AC and EC treatments shows that inflorescence initiation occurred earlier at EC for nearly all lines even though significantly more leaves were formed at EC. Therefore, alteration of time for inflorescence initiation in transgenic lines occurred independent of leaf number (Figure 2).

**Figure 4:**
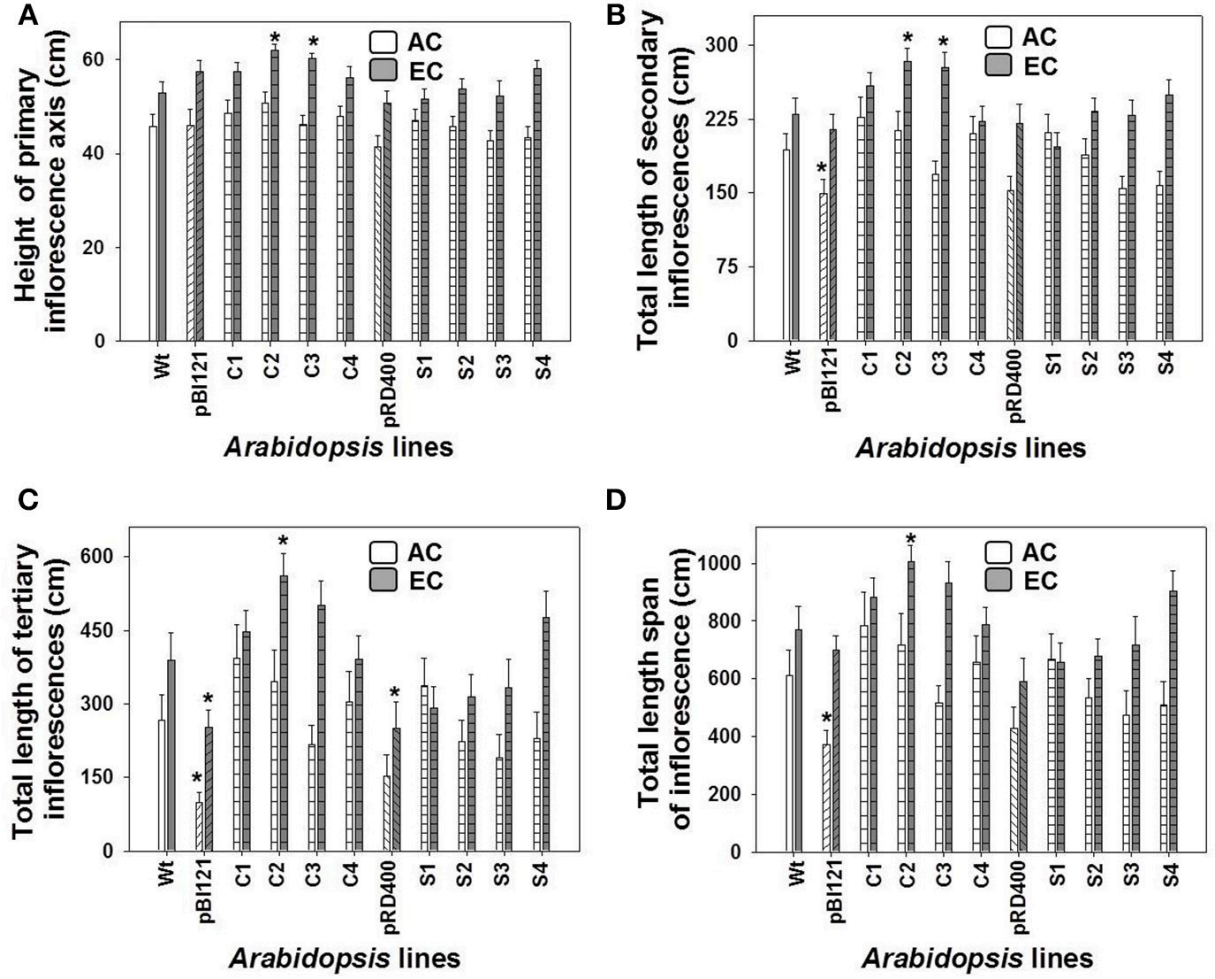
**The lengths of 1°, 2°, and 3° inflorescences and the total inflorescence length in ***Arabidopsis*** lines grown at either AC or EC**. The final height of the 1° inflorescence axis **(A)**, and the total lengths of the 2° **(B)**, 3° **(C)** inflorescences, and the total length span of inflorescence **(D)** for *Arabidopsis* lines grown at either AC or EC are presented. CO_2_ treatments: 380 ppm (AC, white bars), 700 ppm (EC, gray bars). Asterisks (^*^) indicate results significantly different from wild-type (WT) at α = 0.05. Values represent the mean ± SE; *n* = 10 plants per line. Representative plants for each CO_2_ treatment are shown in Supplementary Figure 4.

Growth of the 1° inflorescence showed a sigmoidal growth pattern for all *A. thaliana* lines at AC and EC (Supplementary Figures 3A–D). The height of the 1° inflorescence and total length of 2° and 3° inflorescences, and total length span of the inflorescence were larger in all *A. thaliana* lines at EC compared to AC (Figure 4, Supplementary Figure 4). At maturity, constitutive transgenic lines C2 and C3 showed the tallest 1° inflorescences (up to 17% greater than WT) and total length of 2° inflorescences (up to 21% greater) at EC (Figures 4A,B); these lines also exhibited the highest leaf mtPDH activity (Figure 1A). The total inflorescence length was 30 and 21% larger, respectively, in constitutive transgenic lines C2 and C3, at EC (Figure 4D), which could be attributed to enhanced total lengths of 2° and 3° inflorescences (Figures 4B,C). Inflorescence length in seed-specific lines did not differ from WT either at AC or EC.

##### Silique production

**Figure 5:**
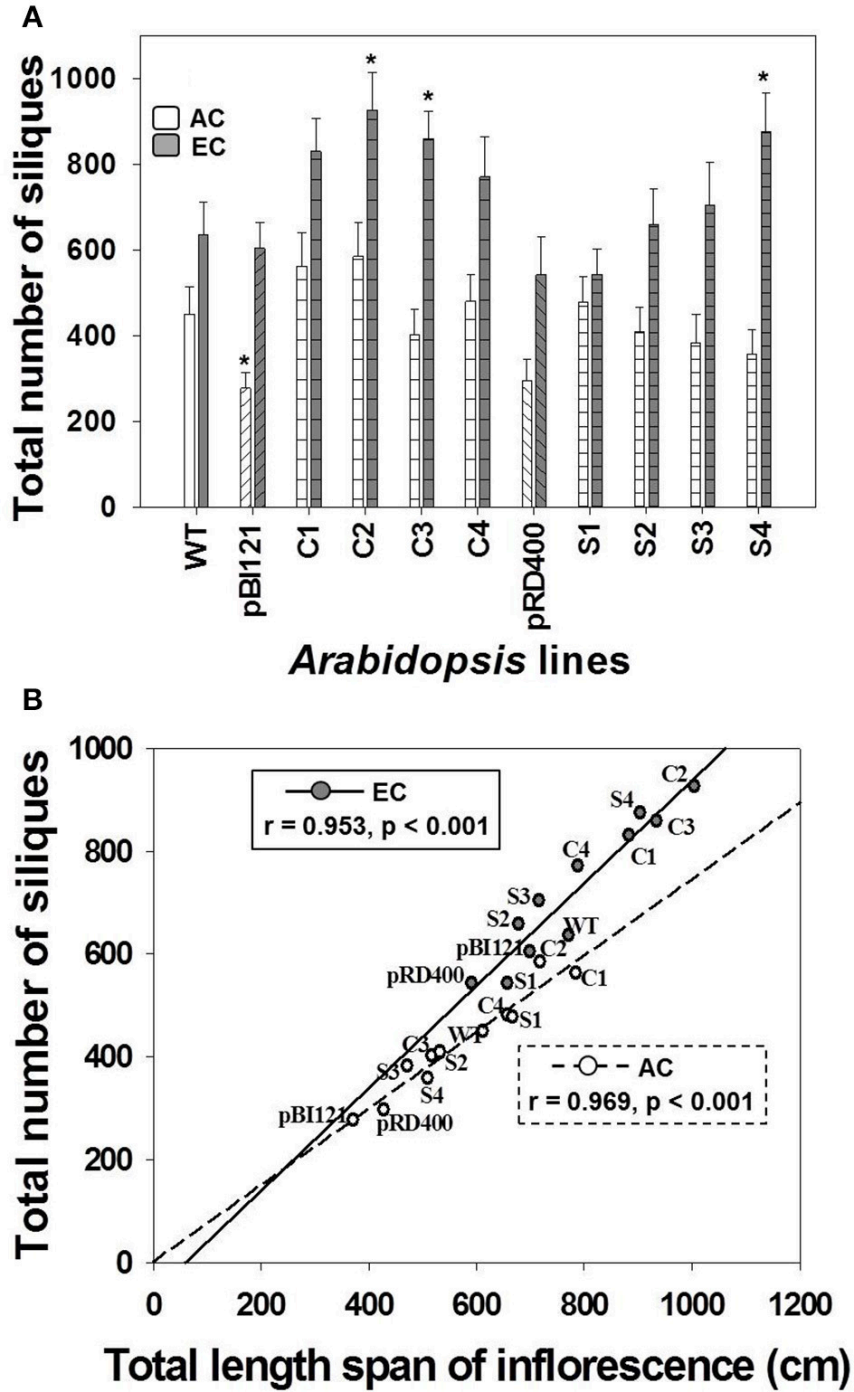
**Silique production in ***Arabidopsis*** lines grown at either AC or EC**. The total number of siliques produced per plant **(A)** and its correlation with total inflorescence length **(B)** is given. CO_2_ treatments: 380 ppm (AC, white bars or open circles/dashed line), 700 ppm (EC, gray bars or closed circles/solid line). Asterisks (^*^) indicate results significantly different from wild-type (WT) at α = 0.05. For **(A)**, values represent the mean ± SE; *n* = 10 plants per line. For **(B)**, the Pearson correlation coefficients (r), significance level, and the lines of best fit are shown.

**Figure 6:**
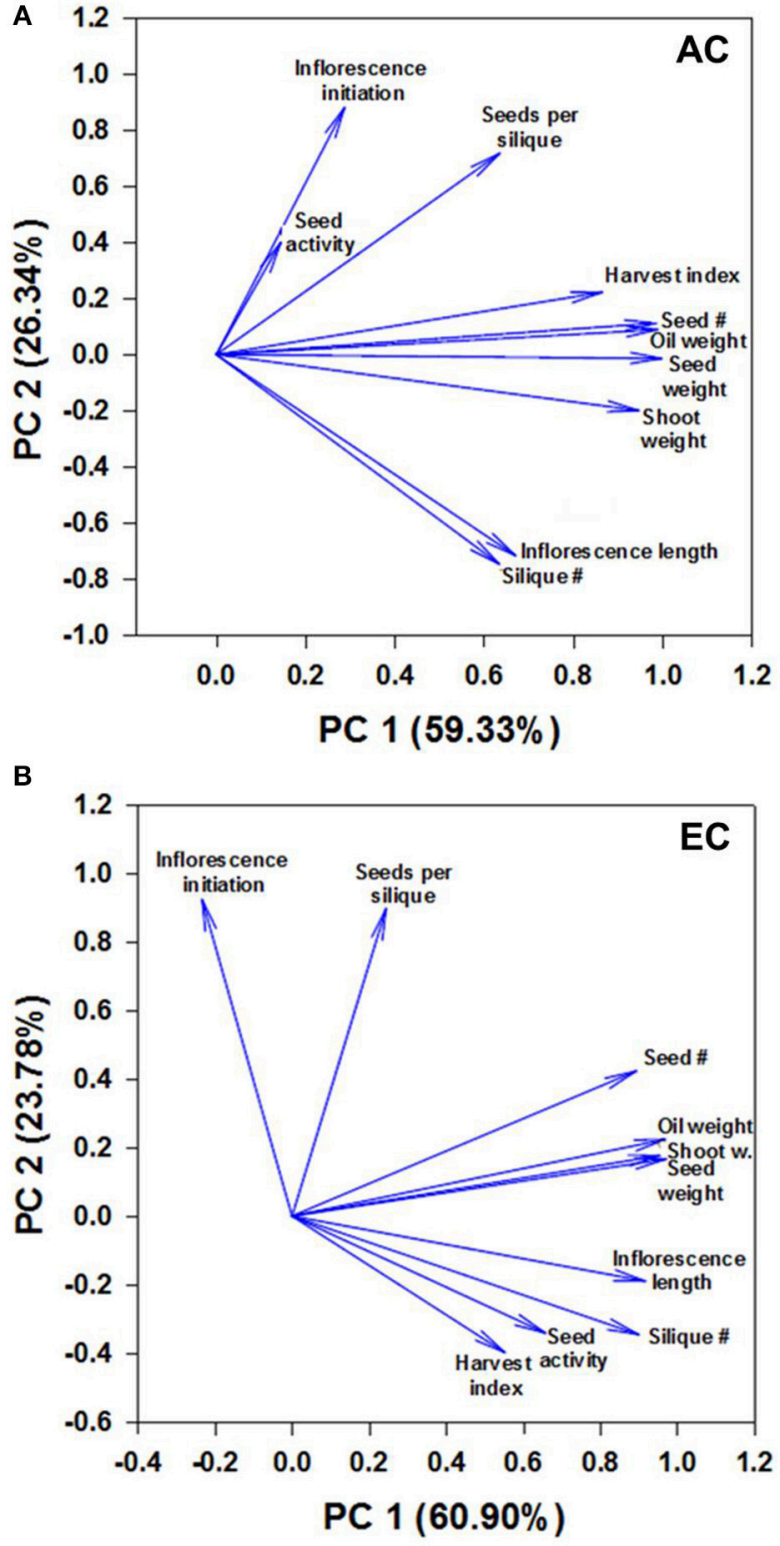
**Component loading plot of growth variables determined by principal component analysis**. Relationships between measured growth characteristics and between these characteristics and seed mtPDH activity at AC **(A)** and EC **(B)** in the space of the two components are presented. PC1 and PC2 are principal components 1 and 2, respectively, and the value within the parenthesis is the percentage contribution of each axis to the total variation. The lengths of the arrows or vectors indicate the strength of the correlations of variables with PC1 and PC2. For plants grown under AC and EC, the component loadings plots showed that the greatest contribution to the values of the first two components was from total seed dry weight, total oil weight, total seed number, and total shoot dry weight as these variables displayed the longest eigenvectors. Smaller the angle between the vectors, stronger the relationship between variables. Moderately large angles between vectors of plotted variables indicate moderate correlations while very large angles between vectors indicate that the corresponding variables are least correlated. The other parameters shown include days to inflorescence initiation, seed mtPDH activity, seed harvest index, 100-seed weight, total inflorescence length and total silique number. The component scores plot is given in Supplementary Figure 5.

In general, there was a significant enhancement in silique production at EC compared to AC (Figure 5A). While a difference in silique production was not found between WT and transgenic lines at AC, constitutive transgenic lines C2 and C3 (with the greatest leaf mtPDH activity and significantly enhanced seed mtPDH activity), and seed-specific line S4 (with the greatest seed mtPDH activity) produced a significantly greater number of siliques than WT at EC (Figure 5A). Silique number at EC in transgenic lines C2, C3, and S4 showed an increase of 46, 35, and 38%, respectively, relative to WT. Total silique number showed a strong positive linear correlation with total inflorescence length both at AC and EC (Figure 5B), and the improvement in silique production in lines C2, C3, and S4 could be attributed to large inflorescences in these lines (Figure 5B). The strong relationship between inflorescence length and silique number was also confirmed by the component loadings plot resulting from the multivariate principal component analysis (Figure 6), which will be discussed in Section *Relationships Amongst Growth Characteristics, and between Growth Characteristics and Seed mtPDH Activity at AC and EC*.

##### Seed production

**Figure 7:**
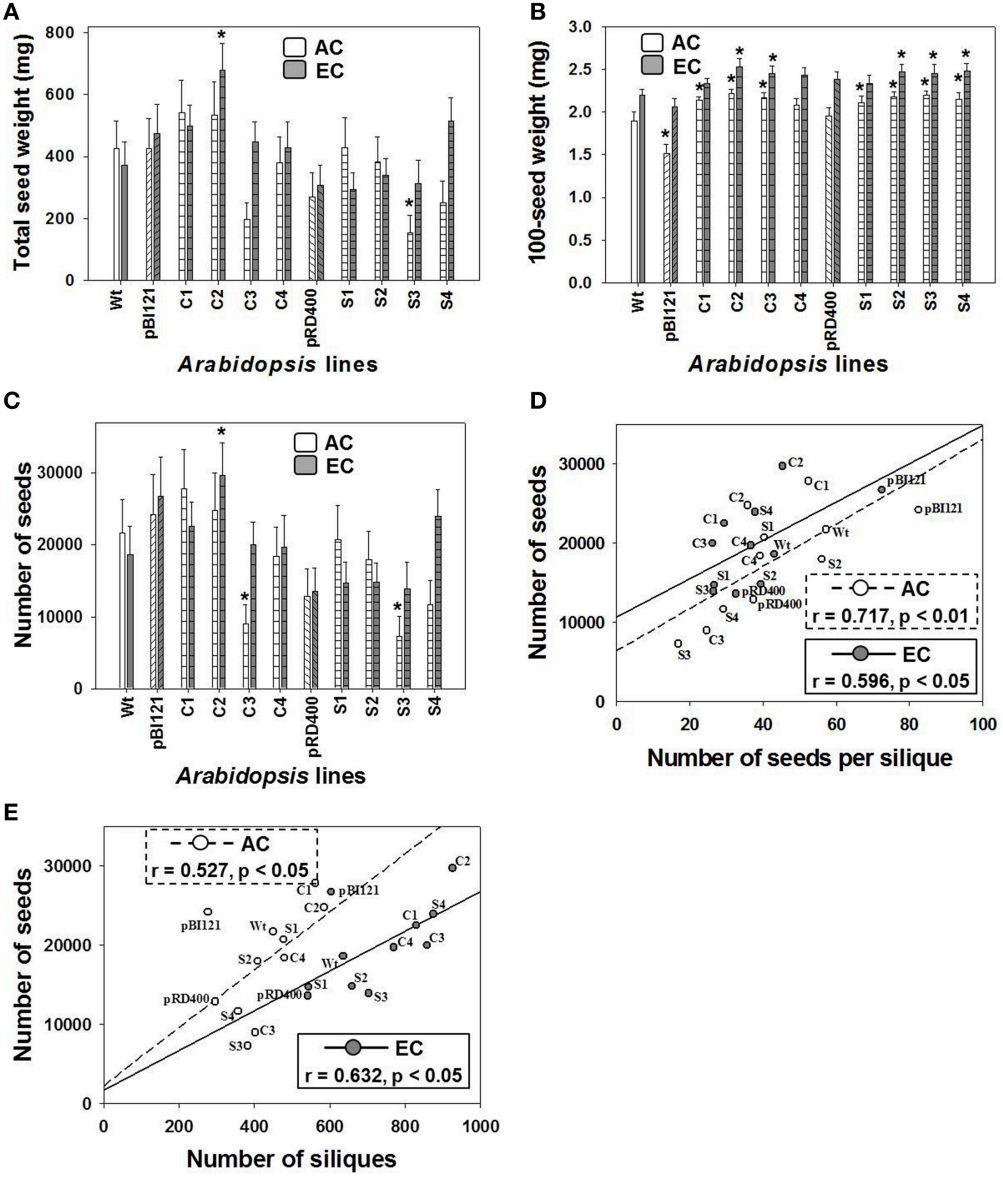
**Seed production in ***Arabidopsis*** lines grown at either AC or EC**. Total seed weight **(A)**, average weight of 100 seeds **(B)**, total number of seeds **(C)** and its correlation with the number of seeds per silique **(D)**, and the total number of siliques **(E)** is provided. CO_2_ treatments: 380 ppm (AC, white bars or open circles/dashed line), 700 ppm (EC, gray bars or closed circles/solid line). Asterisks (^*^) indicate results significantly different from wild type (WT) at α = 0.05. For **(A–C)**, values represent the mean ± SE; *n* = 10 plants per line. For **(D,E)**, the Pearson correlation coefficients (r), significance level, and the lines of best fit are shown.

The total seed weight of constitutive transgenic line C2 having the highest leaf mtPDH activity at EC was 1.8 times greater than WT at EC. This effect was not found at AC (Figure 7A).

At EC a significant increase in 100-seed weight up to 14% was found for constitutive lines C2 and C3, and seed-specific lines S2, S3, and S4 (Figure 7B). At AC, constitutive transgenic lines C1, C2, and C3 and all seed-specific lines showed a significant 10–14% increase in 100-seed weight. In general, 100-seed weight was higher at EC than at AC (Figure 7B). The seed oil weight to seed dry weight ratios were 0.262 ± 0.017, 0.292 ± 0.009, and 0.269 ± 0.012 at AC and 0.301 ± 0.008, 0.310 ± 0.006, and 0.302 ± 0.006 at EC for control lines, constitutive lines and seed-specific lines respectively. The ratios within each CO_2_ treatment were not significantly different from one another. The ratios at EC were significantly larger than the ratio of the controls and seed-specific lines at AC.

Similar to total seed weight, total seed number in constitutive line C2 was enhanced by 2-fold compared to WT at EC (Figure 7C), which largely contributed to the enhancement of total seed weight at EC (Figure 7A). Seed number did not differ between *Arabidopsis* lines at AC (Figure 7C).

Moderate, yet significant positive linear correlations were found between the total number of seeds and the seed number per silique (Figure 7D), and also between total number of seeds and siliques (Figure 7E), at both AC and EC. These data show that the significant enhancement in the total number of seeds in C2 was a result of both an enhancement in silique number and the number of seeds per silique. This moderate relationship was also confirmed by the component loadings plot resulting from the multivariate principal component analysis (Figure 6).

**Figure 8:**
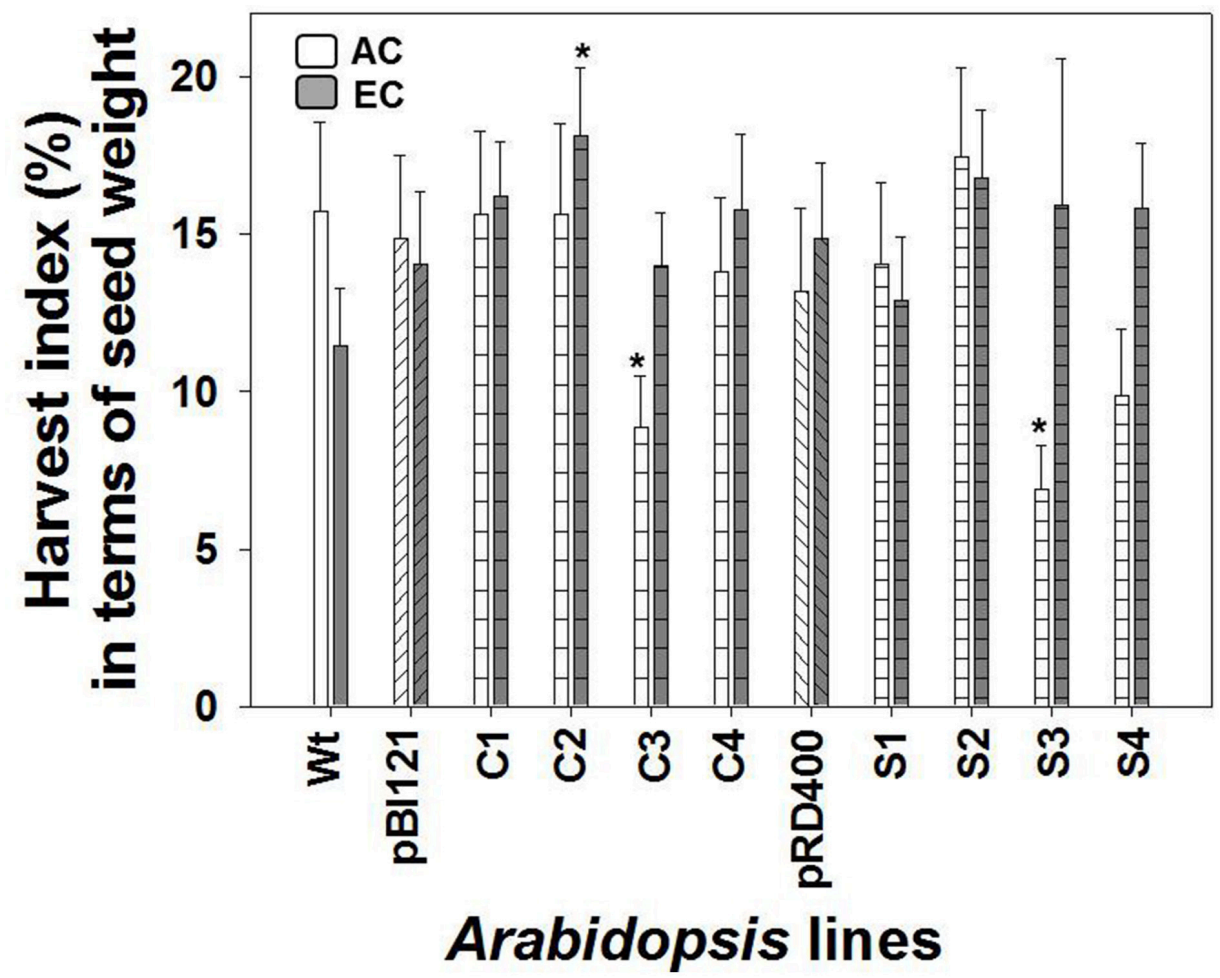
**Harvest index in ***Arabidopsis*** lines grown at either AC or EC**. Harvest index in terms of seed production is presented. CO_2_ treatments: 380 ppm (AC, white bars or open circles/dashed line), 700 ppm (EC, gray bars or closed circles/solid line). Asterisks (^*^) indicate results significantly different from wild type (WT) at α = 0.05. Values represent the mean ± SE; *n* = 10 plants per line.

Owing to its enhanced seed weight, HI in constitutive line C2 was 2-fold greater than WT (Figure 8). HI did not differ between *Arabidopsis* lines at AC (Figure 8).

##### Relationships amongst growth characteristics, and between growth characteristics and seed mtPDH activity at AC and EC

The principal component analysis revealed 3 groups of strongly correlated variables at AC: group 1—seed mtPDH activity, inflorescence initiation, and seeds per silique; group 2—harvest index for seed, total seed number, total oil weight, total seed dry weight, total shoot dry weight; group 3—total inflorescence length, total silique number (Figure 6A). Small angles between the vectors for harvest index, seed number, oil weight, seed dry weight and shoot weight, and between inflorescence length and silique number indicate a significant correlation between these characteristics at AC. Two groups of strongly correlated variables at were identified at EC: group 1—seed number, oil weight, seed dry weight, shoot dry weight; group 2—inflorescence length, silique number, seed mtPDH activity, harvest index (Figure 6B). Inflorescence initiation and seeds per silique correlated least with the other parameters at EC. Transgenic lines C1 and C2 which showed higher inflorescence growth and seed production at AC showed distinct clustering at AC in the component scores plot (Supplementary Figure 5A). Transgenic lines which showed the greatest improvements in inflorescence growth and seed production at EC, clustered together and were clearly separated from the control lines at EC (Supplementary Figure 5B). Correlation analysis further supported a strong linear relationship at EC between seed mtPDH activity, total inflorescence length, and total silique number (Supplementary Figures 6A,B), although there was not a significant linear relationship between HI in terms of seed production and seed mtPDH activity (Supplementary Figure 6C).

## Discussion

### Mitochondrial PDH activity in rosette leaves and immature seeds was enhanced under EC

The present study is the first to show an enhancement of mtPDH activity at EC compared to AC in both in source and sink tissues in *Arabidopsis*, providing direct enzymatic evidence for upregulation in the supply of carbon for mitochondrial metabolism via mtPDH in plants grown at EC (Dahal et al., 2014; Leonardos et al., 2014). These data support recent findings that the glycolytic pathway and/or TCA cycle is enhanced at EC (Leakey et al., 2009; Leonardos et al., 2014). Robertson et al. (1995) showed an increase in mtPDH protein levels in wheat at EC. If this holds true for *Arabidopsis*, the fact that mtPDH activity in WT at AC is not different from EC may be because of mtPDHK suppression. However, elevated mtPDH activity in transgenic lines at EC shows that this repression in WT could be alleviated by antisense suppression of mtPDHK.

The rosette and seed assays developed in the present study facilitated analysis of a large number of samples, and it enabled the identification of a number of *Arabidopsis* lines that show wide variation in leaf and seed mtPDH activities, thus allowing a dosage effect of mtPDH on plant growth to be examined. We did not examine mtPDH phosphatase (mtPDHP) in the present study, but previous work has shown that *in situ* and *in vitro* activity of mtPDHK is significantly greater than mtPDHP activity, and thus changes in mtPDHK activity are most important in regulating mtPDH (Budde and Randall, 1988, 1990). The ATP-inactivation experiments in the present study show that the majority (95%) of mtPDH activity was removed when extracts were incubated with ATP, and that phosphatase inhibitors were only required to remove the remaining 5% of mtPDH activity, providing evidence that mtPDHK activity is greater than mtPDHP activity in extracts.

### An increase in mtPDH activity enhances reproductive growth, sink activity, and plant productivity in *Arabidopsis* at EC

The present study carried out a comprehensive analysis of *Arabidopsis* that included vegetative and reproductive growth, seed oil content, harvest index and net CO_2_ exchange rates under both AC and EC. The suppression of mtPDHK with a subsequent increase in mtPDH activity significantly improved several reproductive growth parameters, particularly at EC. It was originally hypothesized that seed-specific and not constitutive suppression of mtPDHK would result in the greatest increase in growth, since evidence suggested that constitutive suppression of mtPDHK can have an overall negative impact on whole-plant C balance and thus biomass production at AC (Zou et al., 1999; Marillia et al., 2003). For example, at AC, the seed-specific lines show enhanced respiratory CO_2_ release from siliques only, and they tend to show greater seed oil accumulation (Zou et al., 1999; Marillia et al., 2003). However, in the present study the greatest enhancements in inflorescence stem initiation and growth, silique number and harvest index at EC were found using constitutive suppression of mtPDHK. Traits such as inflorescence length and silique number also showed a strong relationship with seed mtPDH activity at EC. The level of stimulation in seed mtPDH activity was comparable between constitutive and seed-specific lines. In addition, only constitutive line C2 showed a significant increase in productivity at EC in terms of seed production, and seed and oil weight. Line C2 exhibited constitutive suppression of mtPDHK combined with the highest stimulation of mtPDH activity in leaves, and an intermediate level of mtPDH activity in the seeds. These data suggest that the tissue-specific pattern of mtPDHK expression can impact on the growth effects found in the present study.

A strong relationship was found between seed mtPDH activity and inflorescence initiation at AC, and between seed mtPDH activity and inflorescence length, silique number and harvest index at EC. Like constitutive lines, seed-specific lines S2 and S3 showed an enhancement of inflorescence initiation at EC, and line S4 that exhibited the highest seed-mtPDH activity also showed a tendency to produce larger inflorescences and it showed a significant enhancement in silique production compared to WT. Although it is generally believed that the napin promoter used in the present study provides seed-specific expression, Chen et al. (2014) showed that the napin promoter not only affects gene expression in seeds but also in flower primordium and pollen in *Brassica*. This finding is consistent with the effects found for inflorescence initiation and silique production in seed-specific lines in the present study which shows that the napin promoter affects expression in reproductive tissues such as the inflorescence meristem and not just the seed embryo. Collectively, the data show that inflorescence growth and silique production and the harvest index are affected by the level of mtPDH activity in reproductive tissues.

It can be assumed that the observed improvements in inflorescence initiation and growth, and silique and seed production in lines showing enhanced mtPDH activity are as a result of the key roles of mitochondrial respiratory processes, and particularly the TCA cycle, in three stages of flower induction and development: (1) transition from the vegetative to generative phase; (2) anther and pollen production; and (3) ovary development (Landschütze et al., 1995; Zou et al., 1999; Marillia et al., 2003; Yui et al., 2003; León et al., 2007; Li et al., 2011). Interestingly, Marillia et al. (2003), reported an increase in citrate synthase, fumarase, and succinate dehydrogenase activities in leaf mitochondria in mtPDHK-suppressed transgenic *Arabidopsis* lines at AC, providing further evidence for enhanced TCA cycle activity in transgenic lines. In comparison to findings of the present study at EC, suppression of other TCA cycle enzyme expression led to a reduction in vegetative and reproductive growth as well as photosynthesis at AC. For example, *Solanum lycopersicum* with suppressed mitochondrial NAD-dependent-isocitrate dehydrogenase showed a reduction in maximum photosynthetic efficiency and starch content in leaves and fruit size and yield at AC (Sienkiewicz-Porzuceka et al., 2010). *S. lycopersicum* with suppressed fumarase gene expression exhibited significant reductions in dark respiratory rates, photosynthesis and starch content in leaves, fruit yield, root yield, whole plant biomass and harvest index at AC (Nunes-Nesi et al., 2007). However, overexpression of these genes and examination of growth under EC has not been reported to date. Collectively, these data confirm the importance of dark respiration and the TCA cycle in regulating reproductive growth.

### An increase in mtPDH activity enhances vegetative growth and source activity in *Arabidopsis* at EC

The rates of photosynthesis at EC were higher than the rates at AC, demonstrating greater source activity at EC for all *Arabidopsis* lines. In addition, photosynthesis of individual rosette leaves in plants containing developing inflorescences (a sink organ) was greater in both constitutive and seed-specific lines at AC and EC relative to controls, providing evidence for reduced negative feedback effects on photosynthesis through enhanced sink activity. In fact, the elevated rates of net C assimilation in the rosette leaves at AC and EC correlated well with enhanced inflorescence initiation. These data are further supported by Li et al. (2011), who showed that overexpression of *Brassica napus* mtPDHK in *Arabidopsis* leads to lower rates of rosette photosynthesis relative to WT at AC.

Although rosettes are responsible for C assimilation during early stages of the life cycle, the inflorescence can sometimes contribute over 90% of the total fixed C under AC and EC (Earley et al., 2009; Leonardos et al., 2014). Importantly, a larger inflorescence under EC as found for the constitutive transgenic lines can provide a larger photosynthetic surface area, thus enhancing light interception and whole-plant photosynthesis during pod and seed development. These data provide evidence that constitutive transgenic lines had a higher potential for whole-plant net C gain than control lines at EC, thus reflecting enhanced source strength.

### Mitochondrial PDH is a viable target site for improving crop productivity at EC

To our knowledge the present study is the first to demonstrate an increase in mtPDH activity at EC in plants, providing evidence for up-regulation of the supply of carbon for mitochondrial metabolism via mtPDH in plants grown at EC. This also illustrates the importance of respiratory processes in maintaining source-sink balance at EC by affecting growth of reproductive organs in plants.

The present study developed a genetic resource to harness the anabolic properties of dark respiration in plants through antisense repression of mitochondrial mtPDHK which results in an increase in mtPDH activity. It was demonstrated that the developmental stage-selective partial repression of mtPDHK (constitutive (ubiquitous) vs. seed-specific) and the degree of alteration in mtPDH activity determine the extent to which plant reproductive growth and oil productivity are enhanced under EC. Collectively, the data show that mtPDHK plays a key role in regulating sink and source activities in *Arabidopsis*, particularly during the reproductive phase.

## Author contributions

SW and MM collectively implemented the experimental design, maintained the plants, and collected all the growth and gas-exchange data. SW designed and carried out the enzyme assays, graphed, and performed the statistical analyses on all data; EM and DT generated the transgenic lines and were involved with the oil analysis. BM, MM, and BG designed the study. SW, BM, and BG wrote the manuscript; all authors discussed the results and commented on the manuscript.

### Conflict of interest statement

The authors declare that the research was conducted in the absence of any commercial or financial relationships that could be construed as a potential conflict of interest.
