# Supplementary material for: Increased mtPDH Activity Through Antisense Inhibition of Mitochondrial Pyruvate Dehydrogenase Kinase Enhances Inflorescence Initiation, and Inflorescence Growth and Harvest Index at Elevated CO2 in Arabidopsis thaliana
Source: Front Plant Sci. 2016 Feb 12;7:95. doi: 10.3389/fpls.2016.00095 (PMC4751281; doi:10.3389/fpls.2016.00095)
Supplement: Supplementary file 1 [file Table1.docx]

| **Multivariate Tests^a^** | | | | | | | | |
| --- | --- | --- | --- | --- | --- | --- | --- | --- |
| Effect | | Value | F | Hypothesis df | Error df | Sig. | Noncent. Parameter | Observed Power^d^ |
| Intercept | Pillai's Trace | .997 | 9677.391^b^ | 14.000 | 340.000 | 0.000 | 135483.471 | 1.000 |
|  | Wilks' Lambda | .003 | 9677.391^b^ | 14.000 | 340.000 | 0.000 | 135483.471 | 1.000 |
|  | Hotelling's Trace | 398.481 | 9677.391^b^ | 14.000 | 340.000 | 0.000 | 135483.471 | 1.000 |
|  | Roy's Largest Root | 398.481 | 9677.391^b^ | 14.000 | 340.000 | 0.000 | 135483.471 | 1.000 |
| CO2treat | Pillai's Trace | .490 | 23.373^b^ | 14.000 | 340.000 | .000 | 327.215 | 1.000 |
|  | Wilks' Lambda | .510 | 23.373^b^ | 14.000 | 340.000 | .000 | 327.215 | 1.000 |
|  | Hotelling's Trace | .962 | 23.373^b^ | 14.000 | 340.000 | .000 | 327.215 | 1.000 |
|  | Roy's Largest Root | .962 | 23.373^b^ | 14.000 | 340.000 | .000 | 327.215 | 1.000 |
| lines | Pillai's Trace | 1.024 | 2.844 | 140.000 | 3490.000 | .000 | 398.152 | 1.000 |
|  | Wilks' Lambda | .298 | 3.192 | 140.000 | 2807.241 | .000 | 361.773 | 1.000 |
|  | Hotelling's Trace | 1.482 | 3.579 | 140.000 | 3382.000 | .000 | 501.119 | 1.000 |
|  | Roy's Largest Root | .843 | 21.003^c^ | 14.000 | 349.000 | .000 | 294.048 | 1.000 |
| CO2treat * lines | Pillai's Trace | .459 | 1.199 | 140.000 | 3490.000 | .058 | 167.855 | 1.000 |
|  | Wilks' Lambda | .618 | 1.213 | 140.000 | 2807.241 | .049 | 138.590 | 1.000 |
|  | Hotelling's Trace | .507 | 1.224 | 140.000 | 3382.000 | .040 | 171.425 | 1.000 |
|  | Roy's Largest Root | .173 | 4.308^c^ | 14.000 | 349.000 | .000 | 60.315 | 1.000 |
| a. Design: Intercept + CO2treat + lines + CO2treat * lines | | | | | | | | |
| b. Exact statistic | | | | | | | | |
| c. The statistic is an upper bound on F that yields a lower bound on the significance level. | | | | | | | | |
| d. Computed using alpha = .05 | | | | | | | | |

**Supplementary Table 1: Results of the Multivariate Test.** In order to determine whether CO_2_ treatment, *Arabidopsis* lines had a significant impact on the measured growth characteristics, all measured data were subjected to a two factor multivariate analysis of variance (MANOVA) test. When considering the Wilks’ Lamda as the multivariate statistic, results of the MANOVA test showed significant main effects for both CO_2_ treatment and line. In addition, there was a significant interaction effect.
