# Supplementary material for: Increased mtPDH Activity Through Antisense Inhibition of Mitochondrial Pyruvate Dehydrogenase Kinase Enhances Inflorescence Initiation, and Inflorescence Growth and Harvest Index at Elevated CO2 in Arabidopsis thaliana
Source: Front Plant Sci. 2016 Feb 12;7:95. doi: 10.3389/fpls.2016.00095 (PMC4751281; doi:10.3389/fpls.2016.00095)
Supplement: Supplementary file 2 [file Presentation1.pptx]

## Slide 1
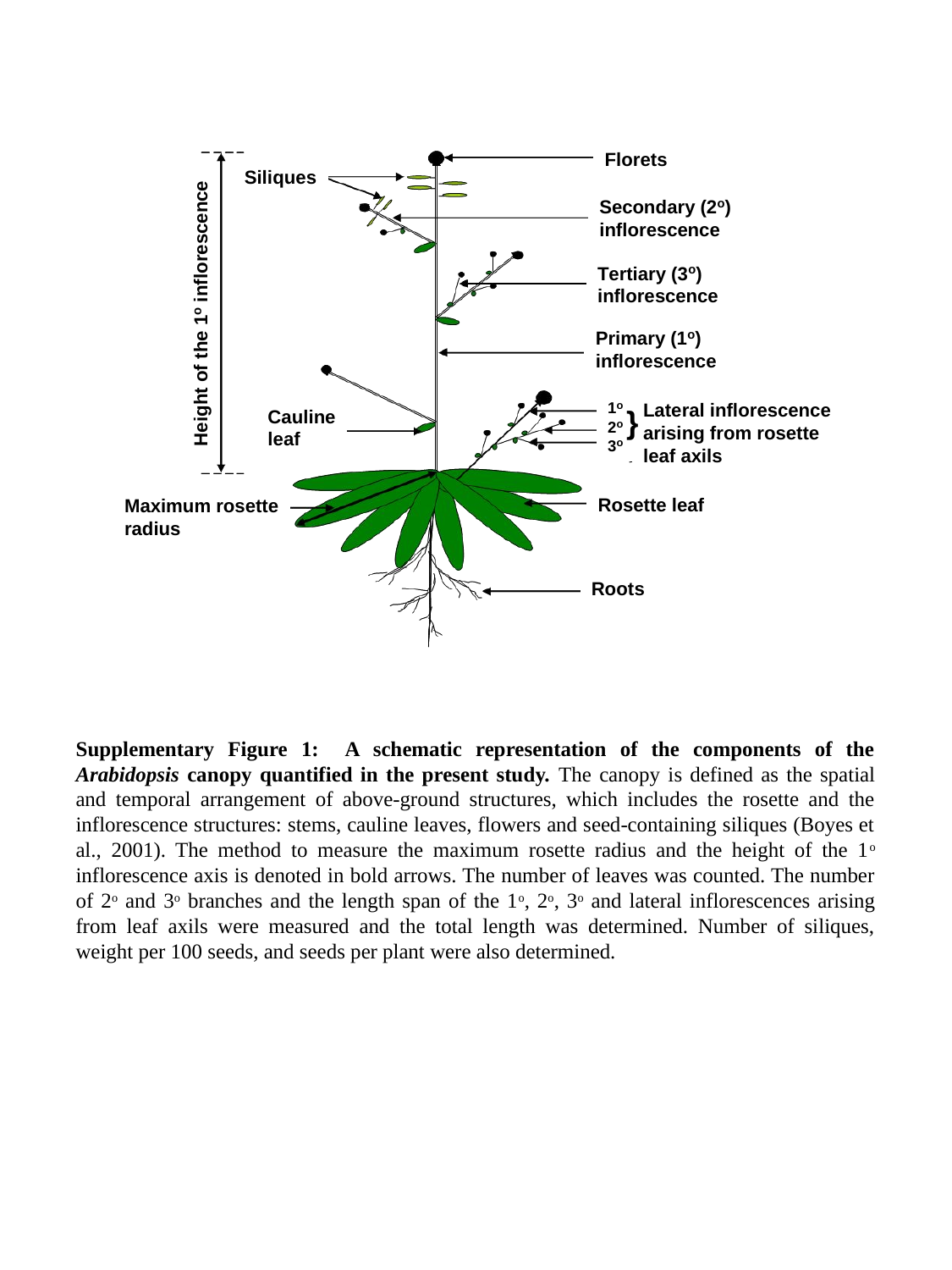

Supplementary Figure 1: A schematic representation of the components of the Arabidopsis canopy quantified in the present study. The canopy is defined as the spatial and temporal arrangement of above-ground structures, which includes the rosette and the inflorescence structures: stems, cauline leaves, flowers and seed-containing siliques (Boyes et al., 2001). The method to measure the maximum rosette radius and the height of the 1o inflorescence axis is denoted in bold arrows. The number of leaves was counted. The number of 2o and 3o branches and the length span of the 1o, 2o, 3o and lateral inflorescences arising from leaf axils were measured and the total length was determined. Number of siliques, weight per 100 seeds, and seeds per plant were also determined.

## Slide 2
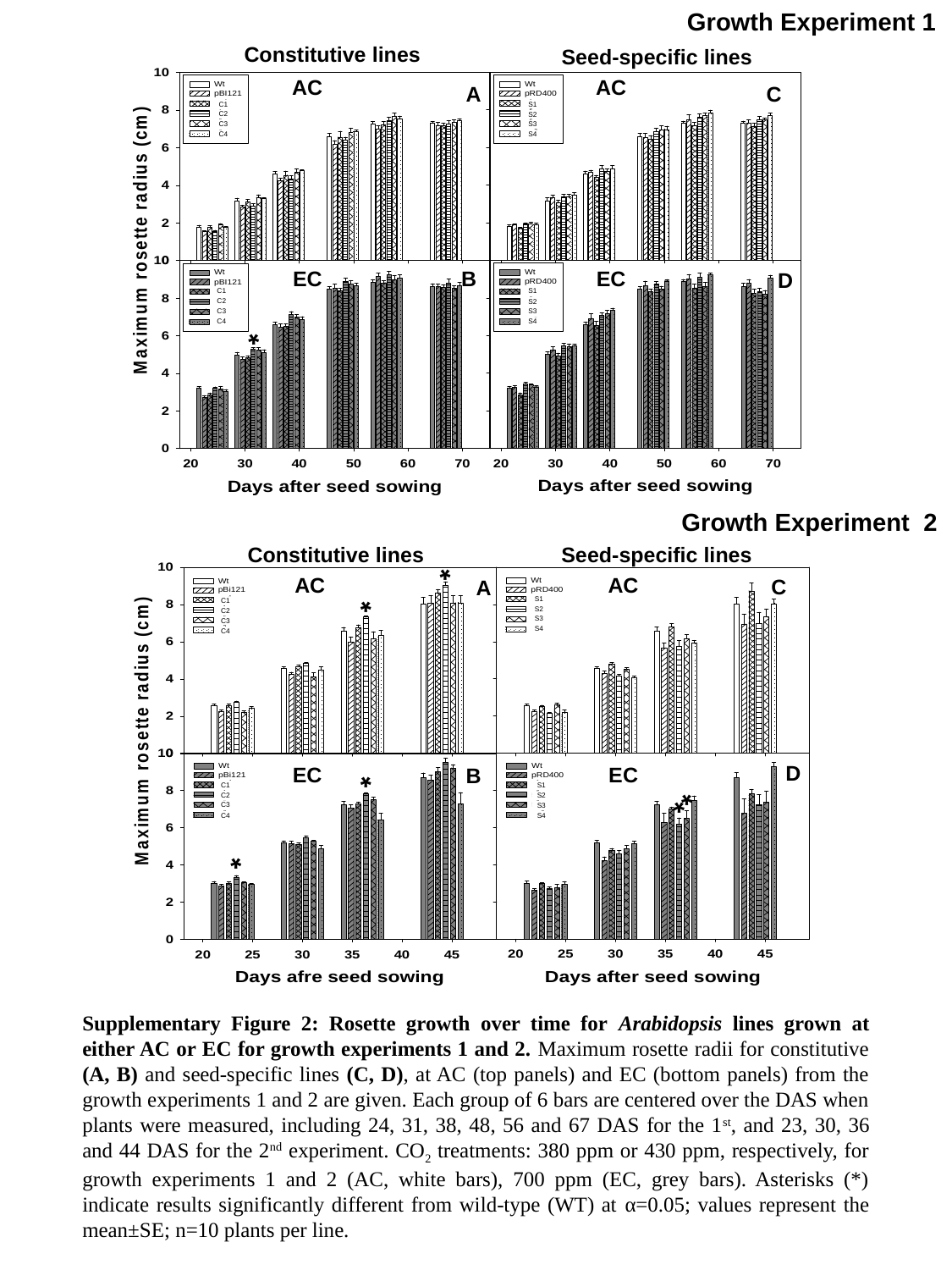

Growth Experiment 1
Constitutive lines
Seed-specific lines
AC
B
D
EC
A
C
AC
EC
*
C1
S1
C2
S2
C3
S3
C4
S4
C1
S1
C2
S2
C3
S3
S4
C4
Growth Experiment 2
Constitutive lines
Seed-specific lines
AC
D
B
EC
*
C
A
AC
*
EC
*
*
*
*
S1
C1
S2
C2
S3
C3
S4
C4
S1
C1
C2
S2
C3
S3
S4
C4
Supplementary Figure 2: Rosette growth over time for Arabidopsis lines grown at either AC or EC for growth experiments 1 and 2. Maximum rosette radii for constitutive (A, B) and seed-specific lines (C, D), at AC (top panels) and EC (bottom panels) from the growth experiments 1 and 2 are given. Each group of 6 bars are centered over the DAS when plants were measured, including 24, 31, 38, 48, 56 and 67 DAS for the 1st, and 23, 30, 36 and 44 DAS for the 2nd experiment. CO2 treatments: 380 ppm or 430 ppm, respectively, for growth experiments 1 and 2 (AC, white bars), 700 ppm (EC, grey bars). Asterisks (*) indicate results significantly different from wild-type (WT) at α=0.05; values represent the mean±SE; n=10 plants per line.

## Slide 3
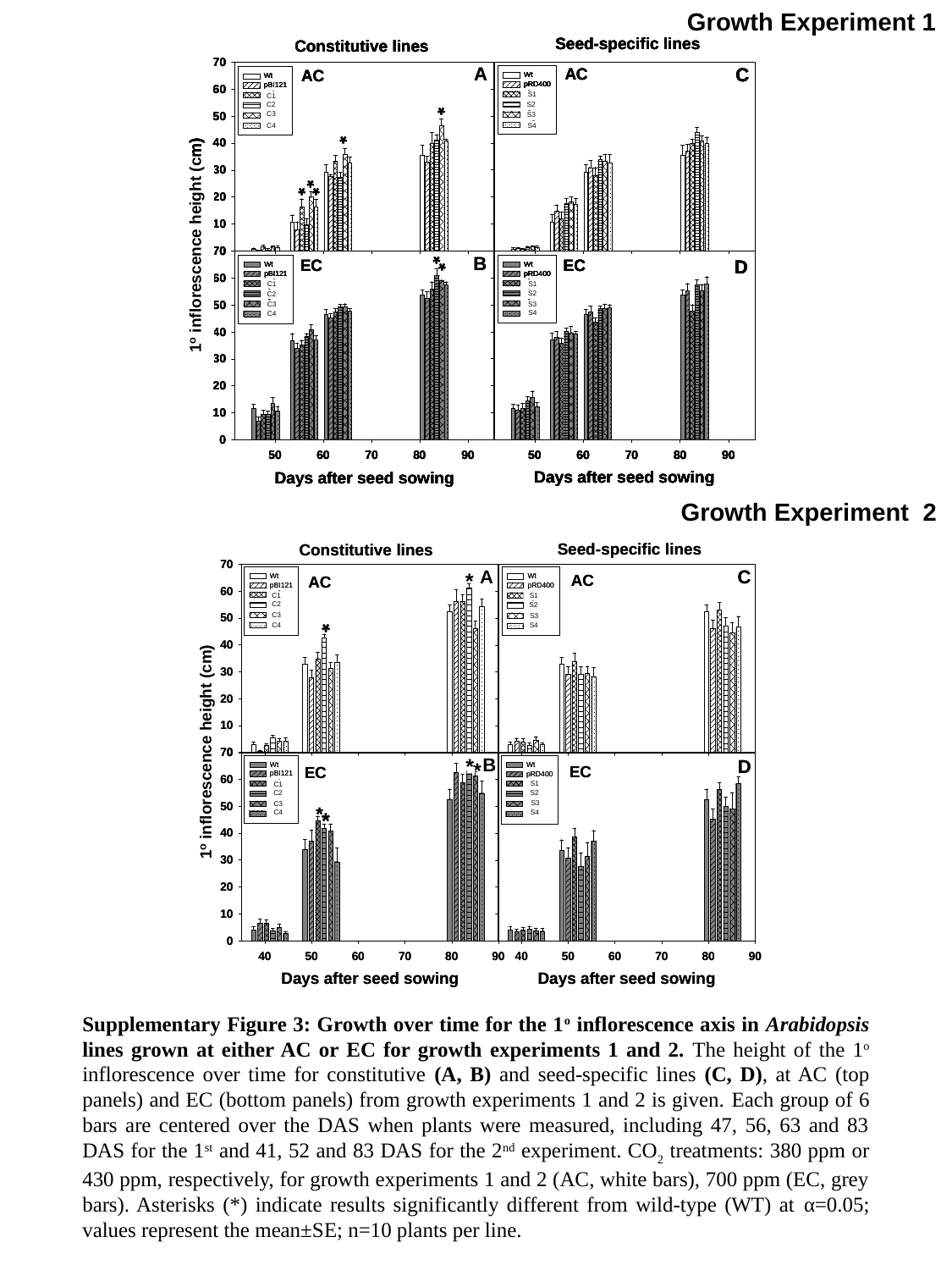

Growth Experiment 1
S1
C1
C2
S2
C3
S3
S4
C4
C1
S1
S2
C2
S3
C3
S4
C4
Growth Experiment 2
S1
C1
C2
S2
C3
S3
S4
C4
S1
C1
C2
S2
S3
C3
C4
S4
Supplementary Figure 3: Growth over time for the 1o inflorescence axis in Arabidopsis lines grown at either AC or EC for growth experiments 1 and 2. The height of the 1o inflorescence over time for constitutive (A, B) and seed-specific lines (C, D), at AC (top panels) and EC (bottom panels) from growth experiments 1 and 2 is given. Each group of 6 bars are centered over the DAS when plants were measured, including 47, 56, 63 and 83 DAS for the 1st and 41, 52 and 83 DAS for the 2nd experiment. CO2 treatments: 380 ppm or 430 ppm, respectively, for growth experiments 1 and 2 (AC, white bars), 700 ppm (EC, grey bars). Asterisks (*) indicate results significantly different from wild-type (WT) at α=0.05; values represent the mean±SE; n=10 plants per line.

## Slide 4
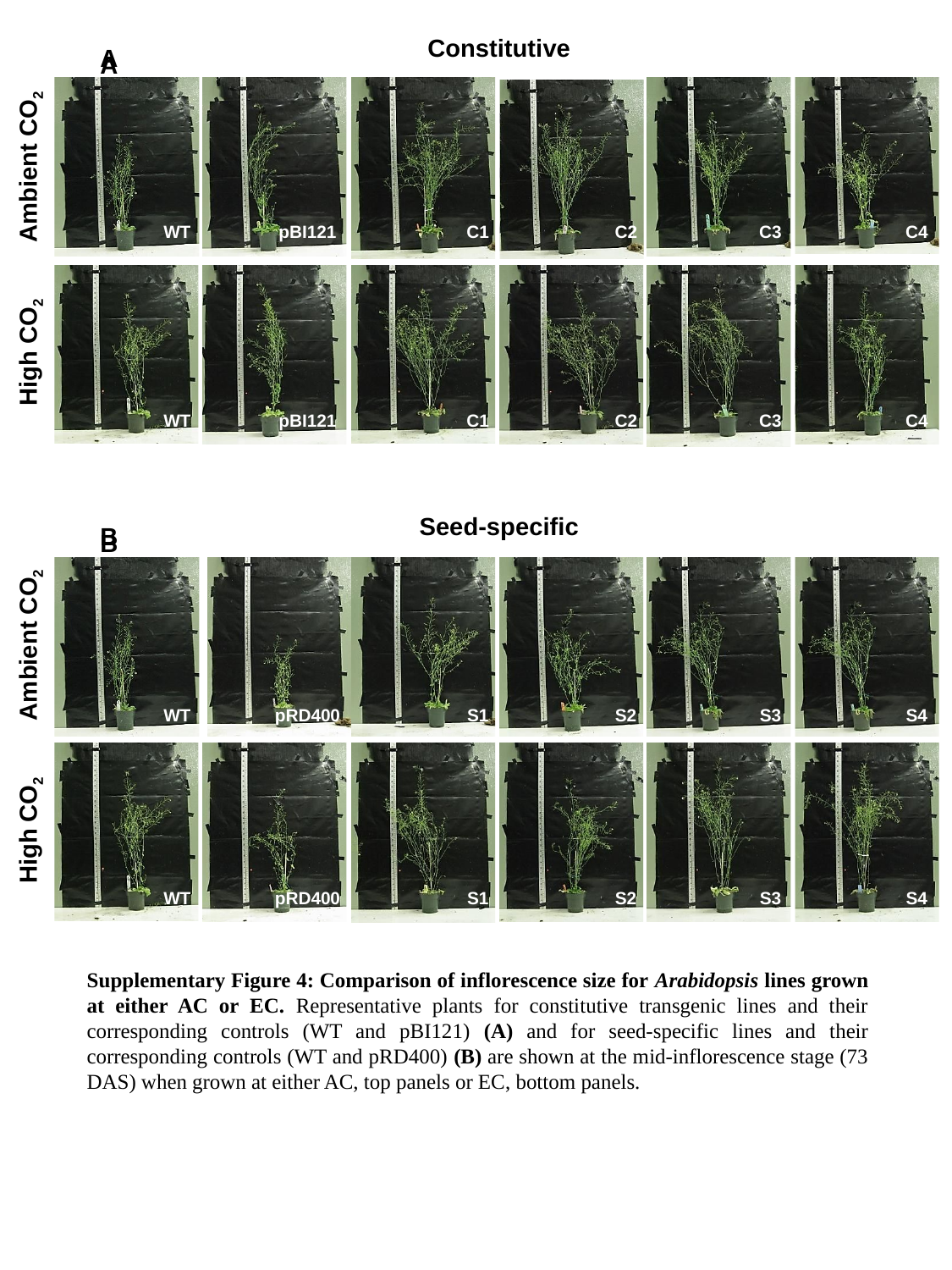

Constitutive
A
A
Ambient CO2
WT
pBI121
C1
C2
C3
C4
High CO2
WT
pBI121
C1
C2
C3
C4
Seed-specific
B
B
Ambient CO2
WT
pRD400
S1
S2
S3
S4
High CO2
WT
pRD400
S1
S2
S3
S4
Supplementary Figure 4: Comparison of inflorescence size for Arabidopsis lines grown at either AC or EC. Representative plants for constitutive transgenic lines and their corresponding controls (WT and pBI121) (A) and for seed-specific lines and their corresponding controls (WT and pRD400) (B) are shown at the mid-inflorescence stage (73 DAS) when grown at either AC, top panels or EC, bottom panels.

## Slide 5
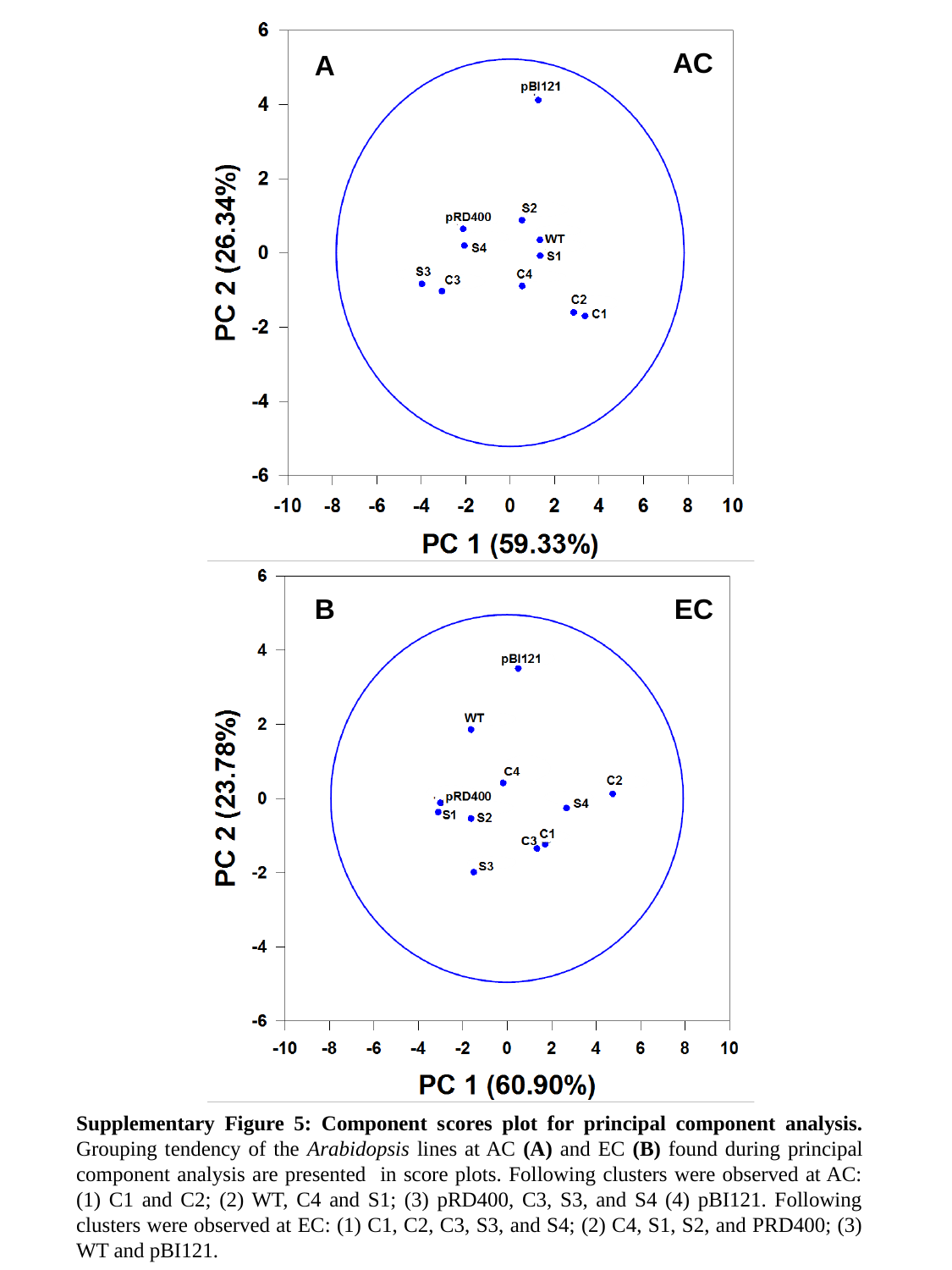

AC
A
EC
B
Supplementary Figure 5: Component scores plot for principal component analysis. Grouping tendency of the Arabidopsis lines at AC (A) and EC (B) found during principal component analysis are presented in score plots. Following clusters were observed at AC: (1) C1 and C2; (2) WT, C4 and S1; (3) pRD400, C3, S3, and S4 (4) pBI121. Following clusters were observed at EC: (1) C1, C2, C3, S3, and S4; (2) C4, S1, S2, and PRD400; (3) WT and pBI121.

## Slide 6
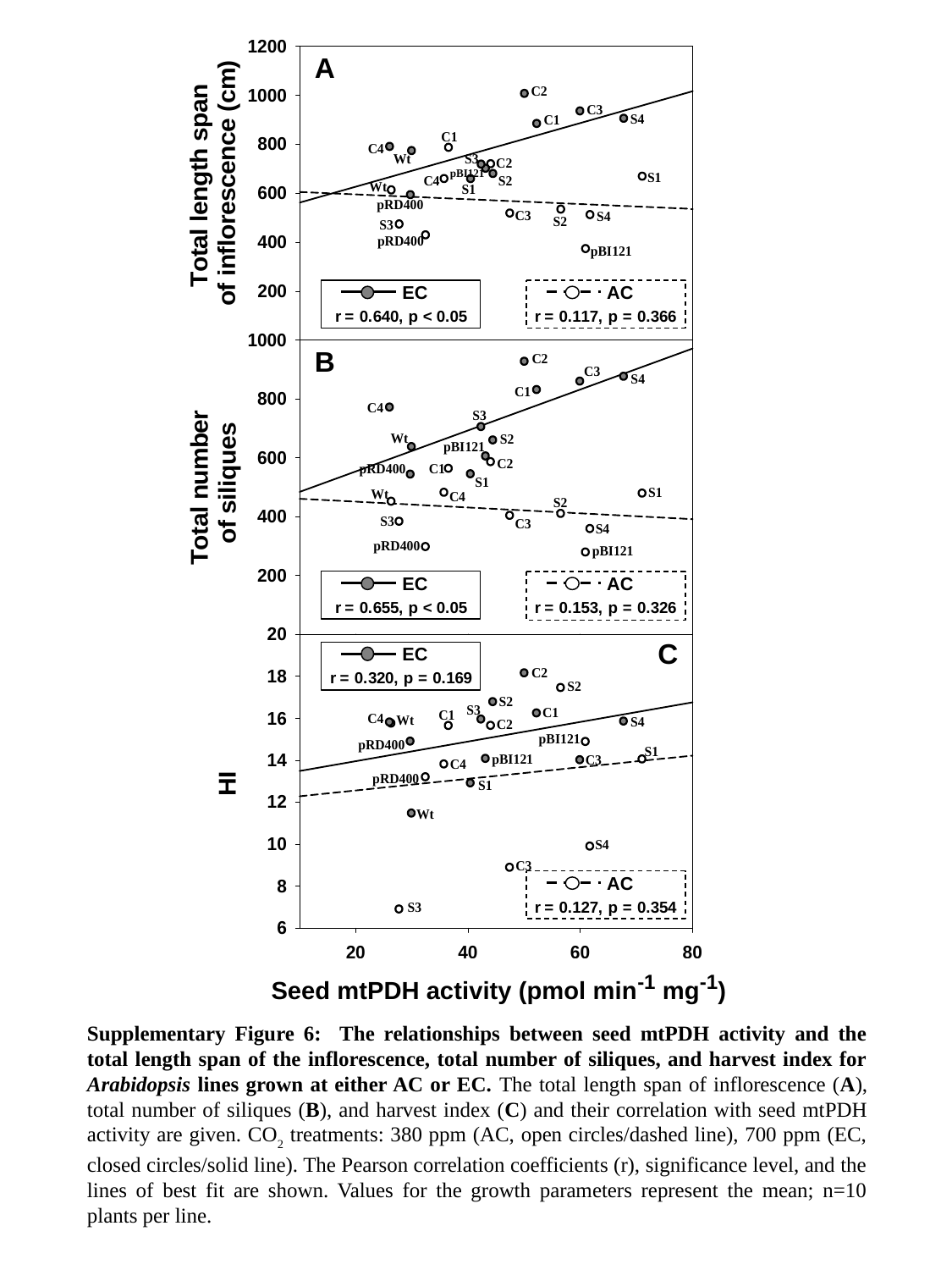

A
B
C
Seed mtPDH activity (pmol min-1 mg-1)
Supplementary Figure 6: The relationships between seed mtPDH activity and the total length span of the inflorescence, total number of siliques, and harvest index for Arabidopsis lines grown at either AC or EC. The total length span of inflorescence (A), total number of siliques (B), and harvest index (C) and their correlation with seed mtPDH activity are given. CO2 treatments: 380 ppm (AC, open circles/dashed line), 700 ppm (EC, closed circles/solid line). The Pearson correlation coefficients (r), significance level, and the lines of best fit are shown. Values for the growth parameters represent the mean; n=10 plants per line.
